# Heteronuclear soliton molecules in optical microresonators

**DOI:** 10.1038/s41467-020-15720-z

**Published:** 2020-05-14

**Authors:** Wenle Weng, Romain Bouchand, Erwan Lucas, Ewelina Obrzud, Tobias Herr, Tobias J. Kippenberg

**Affiliations:** 1 Institute of Physics, Swiss Federal Institute of Technology Lausanne (EPFL), 1015 Lausanne, Switzerland; 2 Swiss Center for Electronics and Microtechnology (CSEM), Rue de l’Observatoire 58, 2000 Neuchâte, Switzerland; 3 Geneva Observatory, University of Geneva, Chemin des Maillettes 51, 12901 Versoix, Switzerland; 4 Present Address: Time and Frequency Division, NIST, Boulder, CO 80305 USA; 5 Present Address: Department of Physics, University of Colorado, Boulder, CO 80309 USA

**Keywords:** Microresonators, Frequency combs, Micro-optics, Solitons

## Abstract

Optical soliton molecules are bound states of solitons that arise from the balance between attractive and repulsive effects. Having been observed in systems ranging from optical fibres to mode-locked lasers, they provide insights into the fundamental interactions between solitons and the underlying dynamics of the nonlinear systems. Here, we enter the multistability regime of a Kerr microresonator to generate superpositions of distinct soliton states that are pumped at the same optical resonance, and report the discovery of heteronuclear dissipative Kerr soliton molecules. Ultrafast electrooptical sampling reveals the tightly short-range bound nature of such soliton molecules, despite comprising cavity solitons of dissimilar amplitudes, durations and carrier frequencies. Besides the significance they hold in resolving soliton dynamics in complex nonlinear systems, such heteronuclear soliton molecules yield coherent frequency combs whose unusual mode structure may find applications in metrology and spectroscopy.

## Abstract (web summary)

Molecules of solitons provide insight into fundamental interactions between them and the underlying nonlinear system. The reported heteronuclear molecules, comprised of dissipative solitons with distinct frequencies, temporal widths, and energies enter the multistability regime and yield in interlocked frequency combs.

## Introduction

Solitons are one of the most fascinating phenomena in nonlinear dynamics due to their universal spatial or temporal localisation of wave forms^1,2^. The ubiquity of solitons has been manifested by the observations in hydraulics^3^, plasmas^4^, semiconductor structures^5^, lasers^6^ and Bose–Einstein condensates^7,8^, despite the difference in the governing nonlinear wave equations. First discovered in optical fibres^9^, optical temporal solitons have also been observed in systems where external driving sources are presented^10^. In these systems, solitons correspond to specific solutions of spatiotemporal self-organisation, which result from a double balance of loss and gain, as well as dispersion and nonlinearity. One specific example is dissipative Kerr solitons (DKSs), which can form in a continuous wave (CW)-driven Kerr cavity^11,12^, as mathematically described by Lugiato–Lefever equation (LLE)^13,14^. With the frequency combs (also referred to as microcombs^15^) they generate, DKSs have been successfully applied in spectroscopy, ranging and telecommunication^16–21^.

Like their spatial counterparts^2,22,23^, temporal solitons can form bound pairs or groups, akin to molecules. Temporal soliton molecules have been observed in conservative systems such as optical fibres^24–27^ and have also been theoretically and experimentally investigated in dissipative systems^28,29^. Moreover, recent advances in dispersive-Fourier-transformation-based imaging techniques have revealed the formation of soliton molecules in a variety of mode-locked lasers^30–34^. Investigation on soliton molecules provides a direct route to study the interactions between solitary waves, and the formation and dissociation of soliton molecules are closely related to subjects such as soliton collision^35^, soliton splashing^36^, soliton rains^37^ and the trapping of solitons^38^. Besides the significance they bring to the fundamental understanding of soliton physics, soliton molecules also present the possibility of transferring optical data surpassing the limitation of binary coding^39,40^.

To date, binding of DKS has only been realised when dispersive waves interlock multiple identical solitons^41–43^, which leads to the formation of “homonuclear” soliton molecules. In addition, except a few special cases (e.g. the soliton crystals reported in ref. ^42^), the inter-soliton separations in homonuclear soliton molecules are generally much larger than the widths of solitons. In this work, we generate heteronuclear DKS molecules, which are stable bound states comprised of solitons with distinct carrier frequencies, temporal widths and soliton energies. This is achieved by pumping one resonance with a laser that is phase-modulated at a frequency that is only one-thousandth of the cavity free spectral range (FSR). This pumping scheme allows us to access a multistability regime where multiple distinct microcomb states can coexist. Theoretically, besides the usual dispersive-wave-mediated long-range binding, we predict the unusual binding mechanism that results in the direct interaction between dissimilar solitons in close proximity. Using a dual-comb sampling technique to measure the inter-soliton separation, we show that distinct solitons can indeed form stable bound structures in systems with instantaneous Kerr nonlinear response, despite the fact that the relative phase between constituent solitons is rapidly rotating.

## Results

### Discrete pumping scheme

**Fig. 1:**
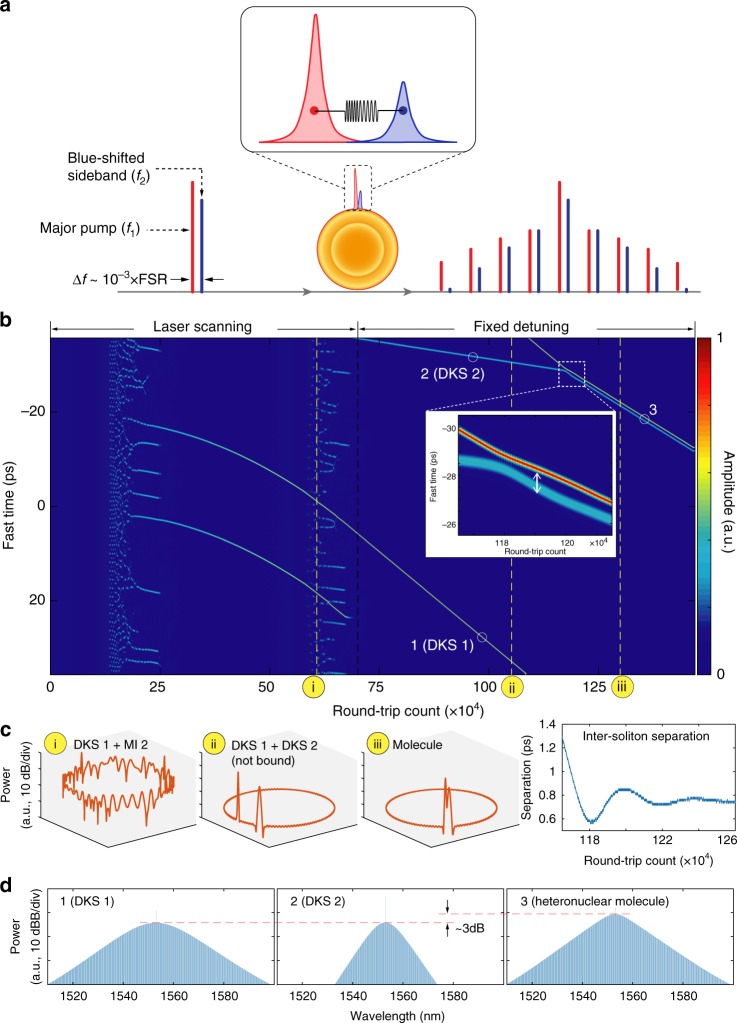
Generation of heteronuclear soliton molecules. **a** Principle of the discrete pumping scheme. Closely bound dissimilar solitons are generated by pumping a single resonance with two laser fields of different frequencies simultaneously. **b** Simulated intracavity field evolution showing the formation of soliton molecules. An enlargement of the soliton binding is shown in the inset. Owing to the periodicity of the fast time axis, the solitons moving out of the map from the bottom will reappear from the top. More details of the simulation can be found in Supplementary Information. **c** Three typical snapshots of the field in **b** at the numbered dashed lines are displayed, corresponding to (1) coexistence of a major soliton and modulation instability; (2) coexistence of unbound dissimilar solitons; and (3) heteronuclear soliton molecule, respectively. The evolution of the inter-soliton separation (indicated by the white double-arrow in the inset in **b**) is presented on the right side, showing the stabilisation of the separation after oscillations. **d** Frequency comb spectra corresponding to the independent solitons pumped by the major pump (major soliton) and by the sideband (minor soliton) as well as the heteronuclear DKS molecules. Comparison of the comb powers shows that the soliton molecule spectrum is the superposition of the spectra of the two dissimilar solitons.

In contrast to a conventional monochromatic pumping scheme, here we drive a single resonance with two laser fields, in order to simultaneously generate two distinct soliton states that are each triggered by the bistability of Kerr cavities. Such complex dynamics of multi-valued stationary states, known as multistability^44^, was recently investigated in fibre ring resonators with a single driving laser pumping two resonances^45^. Naturally, Kerr microresonators appear to be an ideal platform for studying the multistability since they are more robust against environmental perturbations and their strong nonlinearity allows for wider soliton existence range in the frequency domain. However, the small sizes of microresonators lead to very large FSRs that significantly surpass the bistable range, thus forbidding the multistability regime to be entered. Here we demonstrate that such obstacles can be circumvented by driving one resonance with two laser fields. Figure 1a illustrates the concept of pumping one resonance with two laser fields to generate bound states of distinct solitons. Since there is only one resonance being pumped, the LLE model is adequate to describe the dynamics. Including a second pump in the driving term, we express the discrete pumping scheme as:

$$\frac{\partial A}{\partial t}+i\sum_{j = 2}\frac{{D}_{j}}{j!}{\left(\frac{\partial }{i\partial \phi }\right)}^{j}A-ig| A{| }^{2}A=$$

1 $$\left(-\frac{\kappa }{2}+i({\omega }_{0}-{\omega }_{{\rm{p}}})\right)A+\sqrt{{\kappa }_{{\rm{ex}}}}\cdot {s}_{{\rm{in}}}\left(1+\frac{\epsilon }{2}{e}^{i\Omega t}\right)$$

where *A* is the envelope of the intracavity field, *ω*_0_ and *ω*_p_ are the angular frequencies of the pumped resonance and the laser, respectively, *D*_*j*_ is the *j*th order dispersion, *ϕ* is the co-rotating angular coordinate that is related to the round-trip fast time coordinate *τ* by *ϕ* = *τ* × *D*_1_ (where $\frac{{D}_{1}}{2\pi }$ is the FSR), *κ* is the cavity decay rate, *κ*_ex_ is the external coupling rate and $| {s}_{{\rm{in}}}{| }^{2}=\frac{{P}_{{\rm{in}}}}{\hslash {\omega }_{0}}$ is the driving photon flux, where *P*_in_ denotes the power of the main pump. Here $g=\frac{\hslash {\omega }_{0}^{2}c{n}_{2}}{{n}^{2}{V}_{0}}$ is the single photon-induced Kerr frequency shift, where *n* and *n*_2_ are the refractive and nonlinear optical indices, respectively, *V*_0_ is the effective mode volume, and *c* is the speed of light. Moreover, *ϵ* is the modulation index for generating the blue-shifted sideband from a phase modulator, and $\frac{\Omega }{2\pi }$ represents the sideband offset frequency, which is set to be positive to reflect the blue-shifted frequency.

Our theoretical analysis shows that the discrete pumping can simultaneously generate two different soliton states, which can be approximated by the superpositions of solitons excited by only the main pump and by only the sideband, respectively (see Supplementary Notes 1–3 for details). Here we note that our discrete pumping scheme is fundamentally different from bichromatic pumping methods investigated in previous studies^46–49^. First, while the second pumps in previous works were all offset from the main pump laser by approximately one or multiple FSRs, the offset we apply here is only 12–30 MHz, i.e. approximately a thousandth of the FSR (14.09 GHz). Second, the bichromatic pumping scheme was used for modulating the intracavity CW background to manipulate the spatiotemporal characteristics of the otherwise ordinary monochromatically pumped microcombs. In contrast, the modulated laser in this work generates two dissimilar sets of microcombs, each of which would still exist in the absence of the other’s drive.

The simulated formation of heteronuclear soliton molecules is presented in Fig. 1b. As the two pumps are swept towards lower frequencies across a resonance, the major pump excites modulation instability at first, followed by DKS formation. Next, the minor pump scans across the same resonance to generate its microcombs while the major pump is supporting the major DKS. After the minor solitons are formed, due to self-frequency-shifting effects such as high-order dispersion, the major and the minor DKSs travel with different group velocities, until the two solitons are close, with a separation where an equilibrium is achieved. Such equilibrium is obtained when the soliton group velocity difference is balanced with the inter-soliton “repulsion” caused by cross-phase modulation (XPM) (see Supplementary Notes 4 and 5 for further numerical analysis). Intuitively, in Kerr microresonators where the nonlinear response is instantaneous and local, one may expect that discretely driven solitons cannot bind because the Kerr-nonlinearity-mediated effect^25^ averages out as the relative phase between two distinct solitons is constantly rotating, given that the solitons have different carrier frequencies. Indeed, a fixed phase difference between similar solitons (and their oscillatory tails) is essential to the formation of multi-soliton long-range bound states^41,43^. Even in a broader perspective that includes spatial solitons, when the relative phase between driving fields is not fixed (e.g. incoherent solitons and vector solitons), bound states can be formed only when the system’s nonlinearity has a non-instantaneous or nonlocal nature^50–52^. In our work, the XPM effect creates a refractive index barrier to stop solitons of different carrier frequencies (and hence difference group velocities) from colliding. As a result, heteronuclear soliton molecules are formed, with a final group velocity that lies in between the native velocities of the two DKS respectively, according to the conservation of momentum (see Supplementary Note 6 for details).

The corresponding frequency comb spectra are presented in Fig. 1d. Because the major and the minor solitons are of different carrier frequencies, the coarsely resolved comb spectra of heteronuclear molecules acquired by an optical spectrum analyser do not show interference patterns that are typical of monochromatically pumped multi-soliton states^53^. Rather, the averaged comb spectrum of the heteronuclear DKS molecules is the linear superpositions of the spectra of the major and the minor DKSs.

### Experimental generation of heteronuclear soliton molecules

**Fig. 2:**
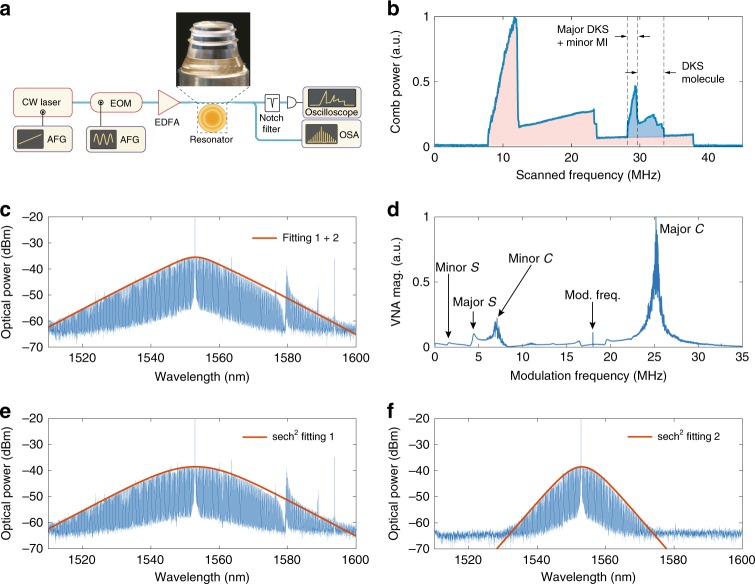
Observation of dissipative Kerr soliton molecules. **a** The schematic experimental set-up. **b** The generated comb power as the laser is frequency swept across a resonance. The red-shaded area is the comb power generated by the major pump, while the blue-shaded area indicates the power of the comb driven by the blue-shifted minor pump. **c** The spectrum of the DKS molecule that is composed of major-single-DKS and minor-single-DKS. **d** The measured response of VNA with modulation probing technique. **e**, **f** Spectra of DKS combs driven by only the major pump and by only the minor pump, respectively. The red traces are the sech^2^ function fittings. The sum of the two fittings is plotted in **c**.

Using an electrooptic modulator (EOM) to produce the minor pump, we generate heteronuclear DKS molecules in a magnesium fluoride (MgF_2_) whispering-gallery-mode resonator (WGMR). The experimental set-up is depicted in Fig. 2a  (see “Methods” for more details). Figure 2b shows the generated microcomb power as the laser frequency sweeps across a resonance. By scanning the laser frequency into the molecule regime, we observe the spectrum of the superposed microcomb of major-single-DKS and minor-single-DKS states (Fig. 2c), while Fig. 2e, f show the spectra of the monochromatically pumped single-DKS states driven by the major and the minor pumps, respectively. Dynamical probing with vector network analyser^54^ measures the radiofrequency (rf) spectrum of the transfer function in Fig. 2d. We observe two sets of the typical double-resonance features that are induced by the soliton resonance (“${\mathcal{S}}$-resonance”) and the CW resonance (“${\mathcal{C}}$-resonance”)^54^, showing that the molecule spectrum is indeed the superposition of two monochromatically driven DKS spectra.

**Fig. 3:**
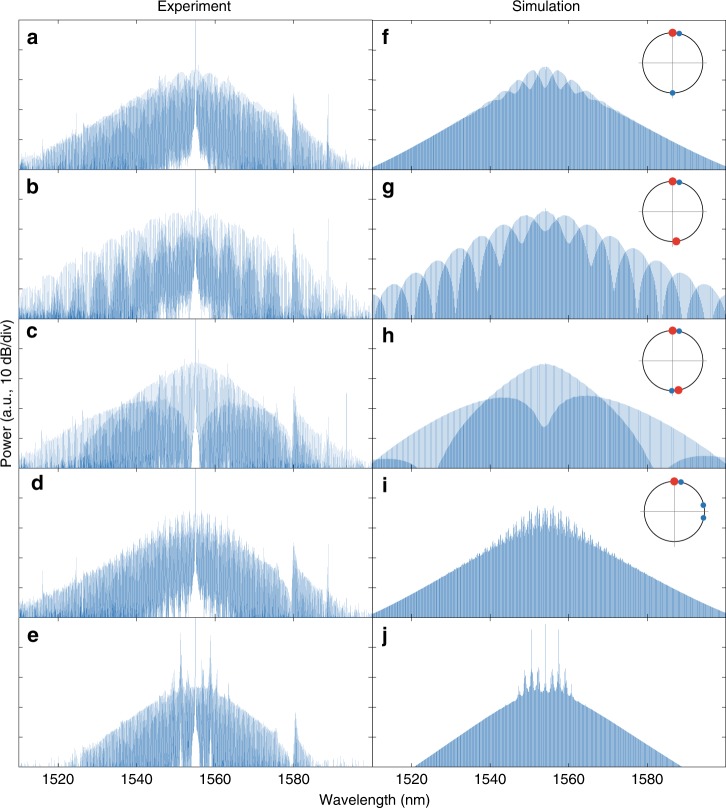
Optical spectra of coexistence of distinct solitons. Experimentally measured (left column) and numerically simulated (right column) spectra of soliton coexistences composed of: **a**, **f** major single-DKS and minor dual-DKS; **b**, **g** major dual-DKS and minor single-DKS; **c**, **h** major dual-DKS and minor dual-DKS; **d**, **i** major single-DKS and minor triple-DKS; **e**, **j** major single-DKS and minor MI. The relative positions of the major solitons (red dots) and the minor solitons (blue dots) are qualitatively depicted on the right side of the simulation panels. For all the simulations, the sideband modulation frequency is set to be 18 MHz and the pump laser detuning is set to be 24 MHz, except for the superposition of major soliton state and minor MI the pump detuning is 17.7 MHz.

We excite a variety of comb patterns of different compositions, which are presented in Fig. 3 with the corresponding simulated spectra, showing remarkable agreement. For all the comb patterns, we observe only one repetition rate, indicating that the coexistences of solitons are truly bound states. Again, we note that the superposed comb spectra do not give information on the separations between the major solitons and the minor solitons in a bound state. This is because stable interference comb spectrum pattern cannot be formed with repetition-rate-synchronised microcombs that have different carrier envelope offset frequencies.

### Structure of soliton molecules

Since the observed comb spectrum indicates that the generated solitons are inevitably impacted by high-order dispersion and mode-crossing-induced dispersive waves, the DKS repetition rate (*f*_rep_) depends on the effective pump-resonance detuning^55,56^. Consequently, the major and the minor DKS should have different *f*_rep_ because of their different detunings (major detuning ${\delta }_{1}=\frac{{\omega }_{0}-{\omega }_{{\rm{p}}}}{2\pi }$ and minor detuning ${\delta }_{2}={\delta }_{1}-\frac{\Omega }{2\pi }$) when one assumes that there is no interaction between them. Such assumption, however, is false. Because the major and the minor solitons share the same optical mode family (i.e. whispering gallery modes with the same polarisation and identical radial and polar numbers), they would either have different *f*_rep_ and show soliton collisions or have the same *f*_rep_ by forming bound states, i.e. DKS molecules. As predicted by our simulation and supported by the single repetition rate of superposed microcombs, the dissimilar solitons travel with same group velocity, likely with a small inter-soliton separation.

**Fig. 4:**
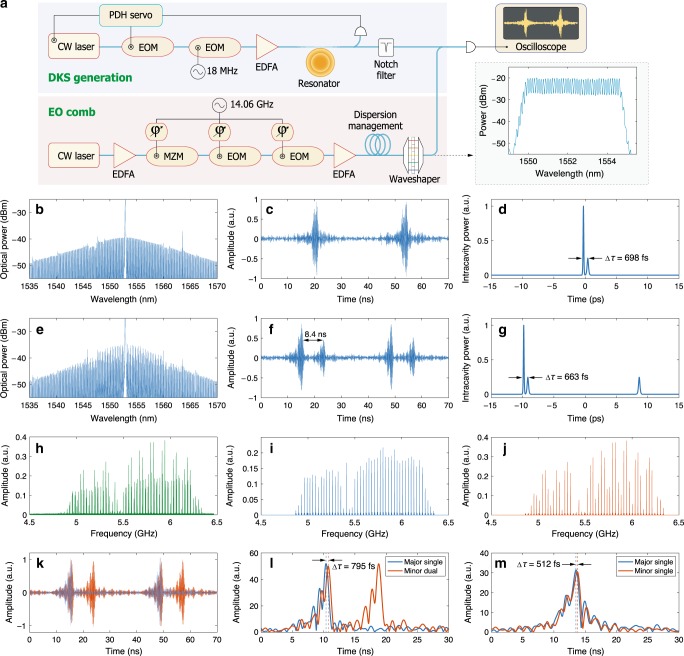
Electrooptic examination of the structures of heteronuclear soliton molecules. **a** The experimental set-up and the optical spectrum of the electrooptic comb. The first EOM serves as the phase modulator for generating PDH error signals in order to lock the pump-resonance detuning. **b**–**g** The optical spectrum (**b**, **e**), the sampled interferograms (**c**, **f**) and the simulated temporal profiles (**d**, **g**) of the major-single-with-minor-single and the major-single-with-minor-dual superposed microcombs, respectively. **h**–**j** The rf spectrum of the interferograms of the major-single-with-minor-dual DKS molecules and the separated spectra of the major DKS and the minor DKS, respectively. **k** The separated interferograms of the microcomb in **e**. **l**, **m** The envelopes (in absolute values) of the separated interferograms of the major-single-with-minor-dual and the major-single-with-minor-single DKS molecules, respectively. The inferred real-time separations between the major DKS and the minor DKS are indicated by double-arrows in **d**, **g**, **l**, **m**.

To verify this prediction, we adopt the imaging technique^36^ with an electrooptic comb (EOC) to examine the bound structures. The set-up is displayed in Fig. 4a. For DKS generation, the pump-resonance detuning is stabilised by implementing the Pound–Drever–Hall (PDH) laser locking technique with an EOM as a phase modulator. The major pump frequency is locked to the high-frequency PDH sideband, thus locking the detuning ($\frac{{\omega }_{0}-{\omega }_{{\rm{p}}}}{2\pi }$) to be equal to the PDH modulation frequency of 25 MHz. The carrier frequency of the EOC is different from the soliton pump laser frequency by  ~5.5 GHz. The repetition rate difference (*Δf*_rep_) between the EOC and the soliton microcombs is set to be  ~30 MHz. As shown in Fig. 4b, c, e, f, the sampled interferograms show only one repetition period, once again showing that the two constituent microcombs have the same *f*_rep_. However, owing to the limited spectral span of the EOC and the chirping of DKS, which is introduced by the notch filter, the interferogram duration is typically >2 ns, which leads to a temporal resolution that is much broader than the inter-soliton separations between dissimilar DKS, thus potentially forbidding us from uncovering the bound structures. Nevertheless, we use fast Fourier transform (FFT) to transform the sampled interferogram streams of the major-single-with-minor-dual DKS microcomb into the rf spectrum (Fig. 4h). Because the major DKS and the minor DKS have different *f*_ceo_, we are able to decompose the rf spectrum into the major-DKS components in Fig. 4i and the minor-DKS components in Fig. 4j. Then we apply inverse FFT to transform the separated rf components back into temporal interferograms to infer the temporal delay between the two soliton streams with resolution that is much shorter than the pulse width of the EOC.

Figure 4k shows the separated interferograms of the solitons whose spectrum is presented in Fig. 4e. The envelopes of the interferograms are shown in Fig. 4l, which also presents the inferred real-time separation between the major and the minor DKS. The same method is also applied to the major-single-with-minor-single soliton streams shown in Fig. 4b, and the separated envelopes are plotted in Fig. 4m. The derived separations of  ~500–800 fs are on the same scale of the temporal pulse width of the individual solitons, providing evidence of extremely short-range binding of distinct solitons. In Fig. 4d, g, we present the simulated molecule profile in the time domain, corresponding to the spectra in Fig. 4b, e, respectively.

### Frequency coherence measurement

**Fig. 5:**
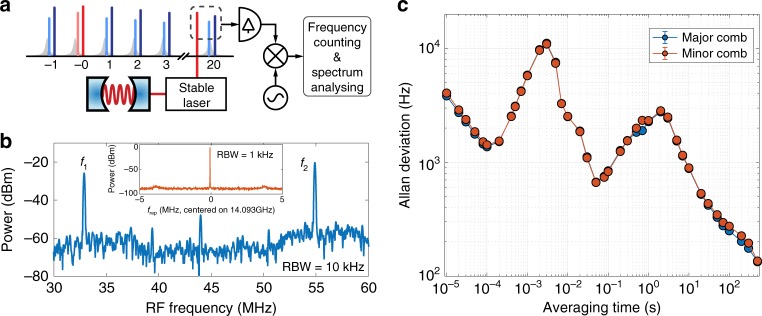
Mutual coherence of soliton molecule. **a** An ultrastable-cavity-locked laser beats with a pair of comb teeth that is 20 FSRs away from the pumped resonance (optical resonances are shown as the grey Lorentzian shapes). The beat frequencies are mixed down to *f*_1_ and *f*_2_ at low frequency range for frequency counting. **b** The rf spectrum of the mixed-down signals, with resolution bandwidth (RBW) of 10 kHz. The frequency difference between *f*_1_ and *f*_2_ is 22 MHz, which is equal to the sideband offset frequency $\frac{\Omega }{2\pi }$ in the experiment. The weak peak at 44 MHz is the second-order harmonic of the EOM modulation frequency. The inset is the repetition rate spectrum of the soliton molecules, showing only one frequency at 14.093 GHz. **c** Calculated Allan deviations of stabilised *f*_1_ and *f*_2_, showing almost identical frequency stabilities. The standard deviation error bars are included but not visible in the figure due to their extremely small amplitudes.

Despite the frequency offset imposed by the driving lasers, the binding of the solitons mutually locks the repetition rates, thus potentially giving rise to a high frequency coherence between the major and the minor DKS microcombs. To test the coherence, we use a 1553-nm laser whose frequency is stabilised to an ultrastable Fabry–Perot cavity to measure the frequencies of a pair of major and minor comb teeth that is 20 FSRs (~2.3 nm) apart from the pumped resonance. The experimental scheme is illustrated in Fig. 5a. The rf spectrum of the beat signals (see Fig. 5b) shows two frequencies that differ by the exact value of the EOM modulation frequency, which was set to be 22 MHz in this experiment. We also fully stabilise the microcomb (see Supplementary Note 7 for the detailed experimental set-up) and then count the two down-mixed beat signals (*f*_1_ and *f*_2_) at the same time and the recorded frequencies allow us to confirm unambiguously that the frequency of the minor comb is offset from the frequency of the major comb by $\frac{\Omega }{2\pi }$. The Allan deviations of the two frequencies are displayed in Fig. 5c, showing almost identical instabilities. We attribute the imperfect overlap of the Allan deviations to the imperfect synchronisation of the counter gating, as well as the fluctuation of the pulse separation in soliton molecules^57^ and the internal motion of soliton molecules^31^. The in-depth analysis of the fluctuations of inter-soliton separations is beyond the scope of this work. Nevertheless, we emphasise here that the frequency coherence the DKS molecule comb exhibited is already sufficient for a wide range of applications in frequency metrology.

## Discussion

We use modulated light to enter a novel multistability regime in a Kerr microresonator to generate heteronuclear soliton molecules. The structures of the soliton molecules, as well as the underlying mechanisms that enable the formation of such DKS bound states, are analysed experimentally and numerically. The mutual frequency coherence of the generated combs is verified with both spectral analysis and frequency counting.

For practical applications, comb-based sensing and metrology may benefit from heteronuclear DKS molecules that provide an additional coherent comb. In particular, with the feature that the major comb and the minor comb are highly coherent despite the fact that they share no frequency components, the heteronuclear solitons can be used with the interlocking of counter-propagating solitons^58^ to generate ultrahigh coherent dual-comb spectrometer without the overlapping of comb teeth (thus no rf spectrum folding). Furthermore, the methods presented in this study may be useful in in-depth investigation of dual-wavelength-pulse synchronisation in mode-locked lasers^59,60^.

## Methods

### Experimental set-up details

The MgF_2_ WGMR is fabricated from a *z*-cut crystalline disk with diamond shaping and surface polishing techniques. The output power of a 1555-nm laser is amplified by an erbium-doped fibre amplifier to  ~300 mW and an EOM is driven by a sine-wave signal output by a function generator to generate optical sidebands. A tapered fibre that is in contact with the WGMR is used to couple light into the resonator. The loaded quality-factor (*Q*) of the mode resonance that generates combs in our work is measured to be  ~1 × 10^9^, corresponding to a resonance bandwidth ($\frac{\kappa }{2\pi }$) of 200 kHz. The transmitted light is filtered with fibre Bragg grating notch filters to suppress the intensive pump light and then registered by a photodetector.

To generate DKS molecules, an arbitrary function generator (AFG) is used to sweep the laser frequency linearly. A sine-wave signal with frequency of 12–30 MHz produced by a second AFG is applied to the EOM to generate optical sidebands of the pump laser. We set the modulation index *ϵ* to be $\sqrt{2}$, i.e. the power of the first-order sideband is half of the main pump power. Once the DKS molecules are generated, because of the self-thermal-stabilisation in the DKS regime, we can maintain the molecule state for up to a few minutes without any active control measures. With active frequency locking applied to the effective detuning, the DKS molecule state can be sustained indefinitely.

### Inter-soliton separation analysis

After the soliton molecules are generated, the microcomb is beaten with the EOC on a fast photodetector to generate interferograms, and the output of the photodetector is recorded by a ultrafast oscilloscope for further analysis.

For the major-single-with-minor-single molecules shown in Fig. 4b, c, the sampling rate of the oscilloscope was set to be 20 GSample/s. Data of 2 ms were recorded, which includes 4 × 10^7^ data points, corresponding to approximately 6 × 10^4^ interferograms. We use the method described in the main text to separate the interferograms of major solitons and minor solitons in the time domain and then locate the positions of maximum intensity of each interferogram to determine the inter-soliton separations. The averaged inter-soliton separation is 512 fs, with a standard deviation of 79.5 fs (15.5% of the separation value).

For the major-single-with-minor-dual molecules shown in Fig. 4e, f, the sampling rate was set to 40 GSample/s, and data of duration of 0.5 ms were recorded, which comprises  ~1.5 × 10^4^ interferograms. Using the same approach, we determine the averaged separation between the major soliton and the minor soliton to be 795 fs, with a standard deviation of 37.4 fs (4.7% of the separation value).

The analysis above shows that the resolution of the electrooptic sampling technique is not limited by the pulse width of the probing EOC and that by increasing the data acquisition rate the accuracy can be further improved. However, one should note that the sampling technique is not capable of accurately characterising the width or the amplitude of individual soliton. Since the EOC has only  ~43 comb teeth, in the frequency domain it can only sample a very limited spectral range of the microcombs. Consequently, while the interferograms can be used to determine the temporal locations of the maximum intensity of solitons, they do not yield the correct pulse widths. And because in the frequency domain the major solitons are more powerful than the minor solitons mostly in terms of the wider spectral span, the central frequency components of the major and the minor solitons sampled by the EOC are very similar in field amplitude. As a result, the amplitudes of interferograms of major solitons and minor ones are almost the same, which is exactly what we observed in Fig. 4k–m.

### Simulation

The LLE-based simulations are performed with adaptive-step-size Runge–Kutta method. For all simulations, the optical mode intrinsic resonance bandwidth is set to be 100 kHz and the critical coupling condition is chosen. To faithfully reproduce the experimental conditions in this work, both the blue-shifted and the red-shifted first-order sidebands generated by the EOM are added to the pump source. In generating the results presented in Fig. 1, artificial perturbations to the intracavity field were made during the laser scanning process in order to obtain the state of single-major-and-single-minor DKS at the end of the scanning. The optical comb spectra of major-DKS-only and minor-DKS-only states are simulated by allowing only the corresponding DKS to exist. The superposed DKS microcomb spectrum is calculated by averaging the immediate comb spectrum for several photon decay times.

## Supplementary information

Supplementary Information

## Data Availability

The data and code used to produce the results of this manuscript are available on Zenodo: [10.5281/zenodo.3668714].
