# Supplementary material for: Heteronuclear soliton molecules in optical microresonators
Source: Nat Commun. 2020 May 14;11:2402. doi: 10.1038/s41467-020-15720-z (PMC7224298; doi:10.1038/s41467-020-15720-z)
Supplement: Supplementary file 1 — Supplementary Information [file 41467_2020_15720_MOESM1_ESM.pdf]

# Supplementary Information for:
# Heteronuclear dissipative Kerr soliton molecules in optical microresonators

Weng and Bouchand et al.

## SUPPLEMENTARY NOTE 1: SIMULATING DKS MOLECULE SPECTRA

In simulating the superposed comb spectra in Figure 3 in the main text, we use seeded field profiles with fixed detunings instead of arbitrary DKS comb patterns that grow from MI and chaotic states during detuning scanning. Supplementary Figure 1 shows the simulated temporal profiles of the intracavity intensity that correspond to the simulated comb spectra presented in Figure 3 in the main text. Second-order dispersion coefficient $\frac{D_2}{2\pi} = 2\,\text{kHz}$ is used, while higher-order dispersion is excluded to maintain the inter-soliton separations exactly as seeded to faithfully reproduce the experimental observations. This approach, unfortunately, eliminates the difference in group velocities of the major and the minor DKS. Nevertheless, since the interference patterns of the optical spectra are determined by the inter-soliton separations between identical solitons (in either major multi-soliton states or minor multi-soliton states) and the inter-soliton separations between dissimilar DKS do not influence the averaged comb patterns at all, this approach allows us to achieve excellent agreement between the experiments and the simulations. Alternatively, we can add single-mode dispersion deviations to the cavity dispersion to simulate the mode-crossing-induced single-mode dispersive waves in order to create binding between identical solitons. Then with third-order dispersion we can show simulated heteronuclear soliton molecules that are composed of distinct solitons and multiple identical solitons (like Figure 4 (g) in the main text). In that case, however, the excellent agreement between the simulated and the measured comb spectra is difficult to achieve, as the temporal separations between identical solitons are difficult to control precisely. The reason is that in the experiments the comb shows multiple mode-crossing-induced dispersive waves that are difficult to accurately describe in the simulation.

For the soliton positions qualitatively illustrated in the insets of Figure 3, we use the results obtained from the investigation on the bound structures so the major and the minor solitons are closely bound.

To simulate the temporal profiles of the soliton molecules shown in Figure 4 in the main text, we add the third-order dispersion coefficient $\frac{D_3}{2\pi} = -4\,\text{Hz}$ to the cavity dispersion. Moreover, we include mode crossing effect for the single-major-with-dual-minor-DKS state shown in Figure 4 (g). The mode crossing effect produces single-mode dispersive wave emission from mode of $u = 30$ (central mode number $u = 0$) that fixes the separation between the two minor solitons.

## SUPPLEMENTARY NOTE 2: SOLITON MOLECULE EXISTENCE RANGES

We use the LLE model to determine the detuning ranges where stable heteronuclear soliton molecules can exist. The effective major detuning ($\Delta_1 = 2\pi\delta_1$) is changed from $40\,\kappa$ to $130\,\kappa$ with a step of $10\,\kappa$ and the effective minor detuning ($\Delta_2 = 2\pi\delta_2$) is tuned continuously to find the critical detuning values beyond which the soliton molecules disintegrate or annihilate.

Supplementary Figure 2 summarises the simulation results. We find that for a fixed major detuning, there is a maximum minor detuning (red circles) beyond which the soliton molecule state will annihilate due to the constituent solitons' annihilation. Therefore there is a minimum value of the sideband offset frequency $\Omega$ to allow for molecule existence. In our experiment we found that the minimum $\Omega$ is around 12 MHz, which agrees relatively well with the simulation (see Supplementary Figure 3). When $\Delta_2$ is below approximately $9.5\,\kappa$, the minor soliton starts to breath, and will eventually annihilate when $\Delta_2$ is below $\sim 8.5\,\kappa$ (blue squares). This behaviour is just like the typical monochromatically pumped soliton breathers with a small detuning. Interestingly, when $\Delta_1$ is above $80\,\kappa$, there is a critical value of minor detuning (yellow diamonds) below which the major solitons and the minor solitons detach from each other but both exist. Consequently, in the blue shaded range the minor and the major solitons coexist but the stable bound states of heteronuclear molecules cannot form anymore. We suspect that the reason is that as the difference between $\Delta_1$ and $\Delta_2$ becomes larger the intrinsic group velocity mismatch is larger, which will eventually overcome the binding effect. However, in the experiment we never observed molecule disintegration.

We also try different third-order dispersion $D_3$ in the simulation and we find that with other parameters (e. g. pump powers, detunings, FSR, etc.) being kept the same as those used for the simulations for the main text, when $|\frac{D_3}{2\pi}| < 4\,\text{Hz}$ stable soliton molecules can always be formed. When $|\frac{D_3}{2\pi}| > 5\,\text{Hz}$ the soliton molecules are not stable anymore as the molecules can disintegrate or one of the solitons will annihilate.

Although we do not intend to explore the full parameter space for soliton molecule existence as the involved parameters are too many, which makes the thorough investigation on this direction beyond the scope of this work, we test with varied pump powers. The simulation results show that when the minor pump power is approximately a factor of 8 higher than the major pump power the major soliton in the molecule annihilates. However, experimentally we find that the minor pump power needs to be kept below $\sim 70\%$ of the major pump power in order to generate soliton molecules. The in-depth study for this observation is to be carried out in the future.

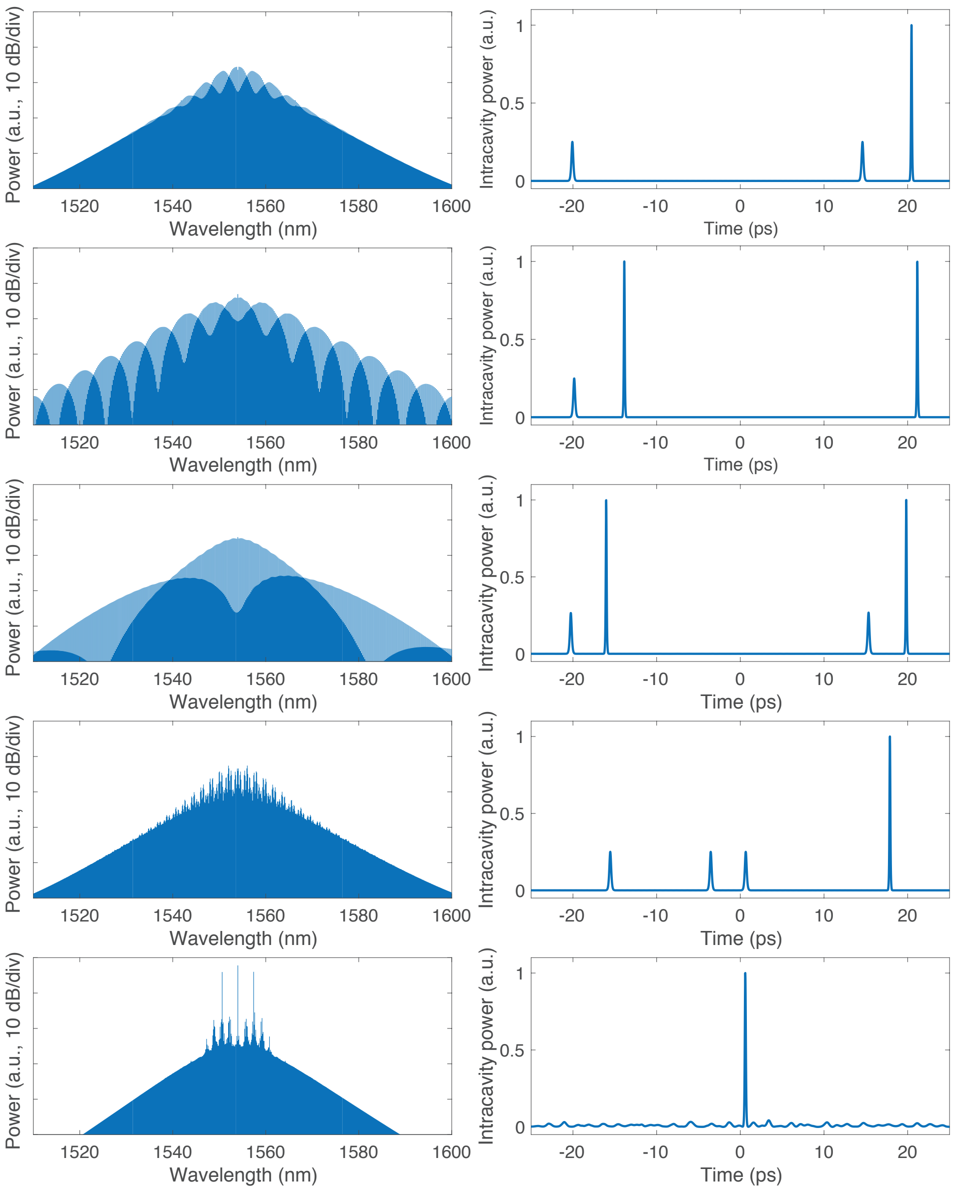


**Supplementary Figure 1**. **Simulated optical spectra of superposed microcombs and the corresponding temporal profiles of the solitons.** Left column: simulated optical spectra of superposed microcombs presented in Figure 3 in the main text. Right column: corresponding temporal profiles of the intracavity intensity.

## SUPPLEMENTARY NOTE 3: DYNAMICAL BRANCHES OF DISCRETELY PUMPED MICROCOMBS

In this section we compare simulations based on discrete pumping with conventional LLE model with monochromatic pumping, and analyse the branches of multistability with the simulation results. We perform numerical simulations based on Eq. 1 in the main text as the main pump and the blue-shifted sideband are swept over an optical mode resonance. The sideband modulation frequency $\Omega$ is fixed to be $90\,\kappa$, and only second-order dispersion $\frac{D_2}{2\pi} = 2\,\text{kHz}$ is included in the cavity dispersion for the simulations. The simulated evolution of the intracavity field amplitude is presented in Supplementary Figure 4 (a), showing the coexistence of major DKS and minor MI state as well as the coexistence of major DKS and minor DKS. Without higher-order dispersion both major and minor DKS have the same group velocity, therefore no heteronuclear DKS molecules are formed due to the lack of restoring force. In Supplementary Figure 4 (b) we plot the peak value of $|A|^2$ of both the major DKS and the minor DKS in green traces. The regimes of coexistence of distinct comb states are marked in the figure too.

We assume that the soliton solutions to Eq. 1 in the main text can be approximated in the form of superpositions

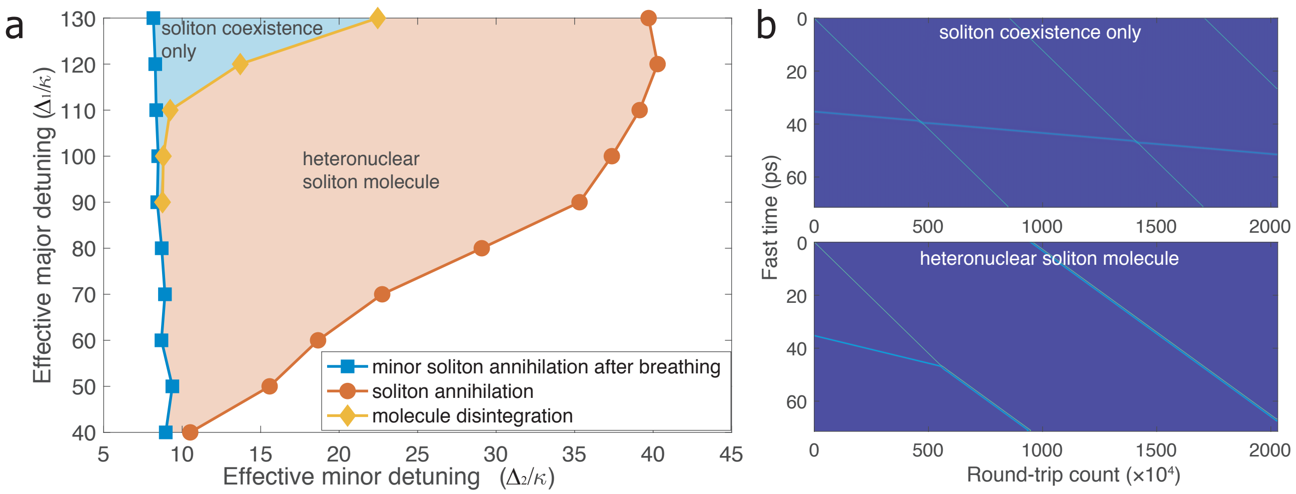


**Supplementary Figure 2**. **Simulated detuning range for the existence of heteronuclear soliton molecules.** (a) The red-shaded range is where stable heteronuclear soliton molecules can exist. In the blue shaded range both the major and the minor solitons coexist, but the bound states of heteronuclear soliton molecules cannot be formed anymore. As a result, the major solitons and the minor solitons travel with different group velocities. (b) Intracavity field amplitude evolutions of two examples: soliton coexistence only (upper figure, major detuning $\Delta_1 = 120\kappa$, minor detuning $\Delta_2 = 10\kappa$) and heteronuclear soliton molecule (lower figure, $\Delta_1 = 120\kappa$, $\Delta_2 = 30\kappa$).

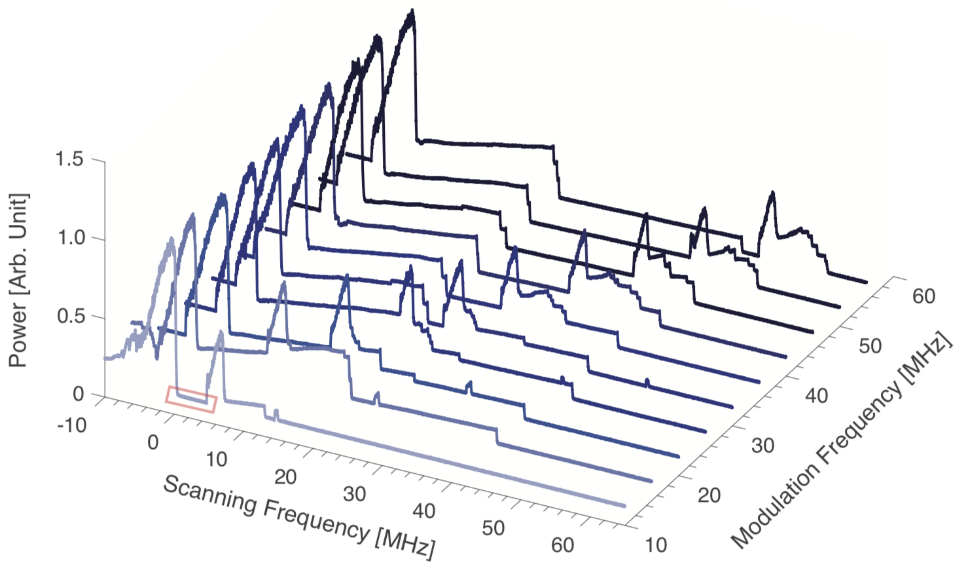


**Supplementary Figure 3**. **Soliton power spectra with different sideband modulation frequencies in soliton molecule generation.** By varying sideband modulation frequency the soliton coexistence (molecule) range is experimentally examined. When the modulation frequency is below $\sim 12\,\mathrm{MHz}$ the major soliton "steps" after the MI stage does not show anymore (indicated by the red box), which means that the minimum $\Omega$ is approximately equal to $60\,\kappa$.

of the soliton solutions to the conventional LLE model driven by only the major pump and by only the sideband respectively, i. e., there is no interaction between dissimilar solitons and to an individual soliton state a driving field of a different frequency is merely a perturbation whose effect can be reasonably ignored. Hence we can express the discrete-pumping scenario with two independent LLEs:

$$\frac{\partial A}{\partial t} - i\frac{1}{2}D_2\frac{\partial^2 A}{\partial \phi^2} - ig|A|^2 A = \left(-\frac{\kappa}{2} + i(\omega_0 - \omega_\mathrm{p})\right) A + \sqrt{\kappa_\mathrm{ex}} \cdot s_\mathrm{in} \tag{1}$$

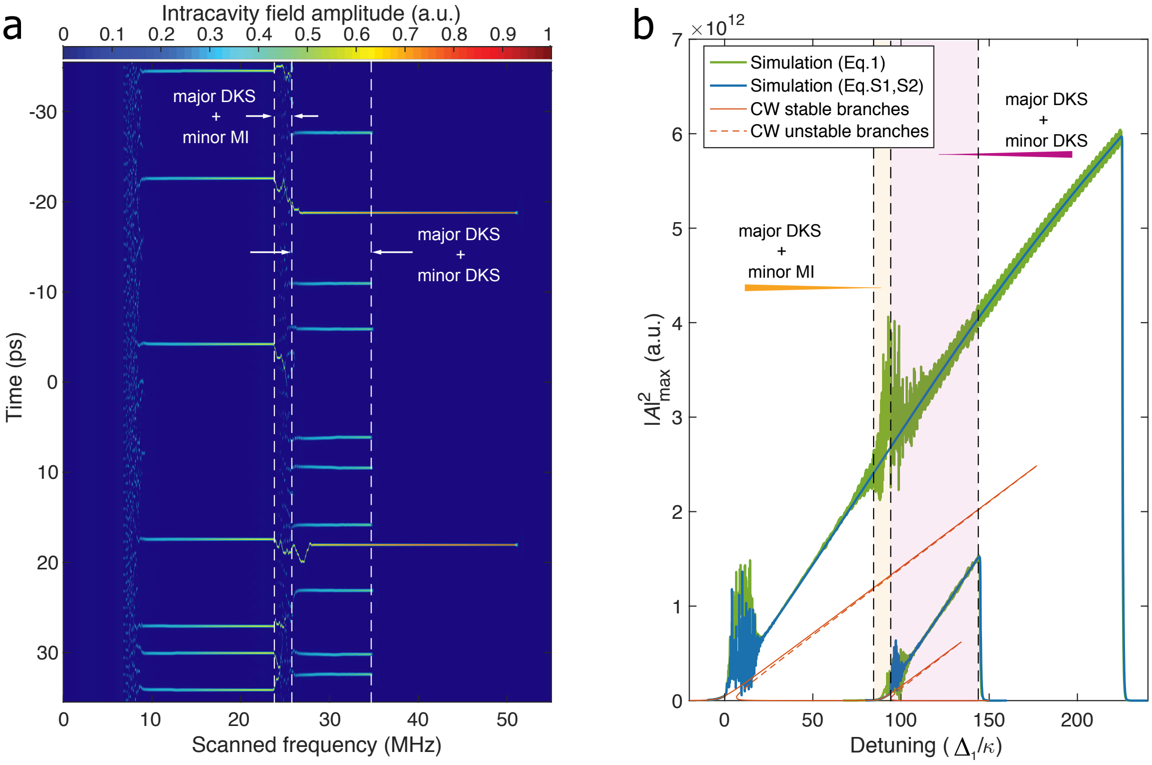


**Supplementary Figure 4. Comparison of numerical simulations based on single LLE with discrete pumping scheme and two independent conventional LLEs.** (a) Evolution of the intracavity field amplitude as the laser frequency is swept across an optical resonance. Between the dashed lines the coexistence of major DKS state and minor MI state appears at first, followed by the coexistence of major DKS (dual-soliton state) and minor DKS (9-soliton state). In the region where the major solitons and minor MI coexist, the locations of major solitons fluctuate heavily and some of them annihilate due to the perturbation from the minor MI. (b) Comparison of simulated $|A|^2_{\mathrm{max}}$ for the major DKS and the minor DKS based on Eq. 1 in the main text and based on Supplementary Equation 1 and Supplementary Equation 2 respectively. The stable (unstable) branches of the CW stead state is also shown in red solid (dashed) curves. Superpositions of major DKS and minor MI can exist in the orange-shaded detuning range and the superpositions of major DKS and minor DKS can be generated in the purple-shaded range.

$$\frac{\partial A}{\partial t} - i\frac{1}{2}D_2\frac{\partial^2 A}{\partial \phi^2} - ig|A|^2A = \left(-\frac{\kappa}{2} + i(\omega_0 - \omega_\mathrm{p} - \Omega)\right)A + \sqrt{\kappa_\mathrm{ex}}\cdot s_\mathrm{sb} \tag{2}$$

where $|s_\mathrm{sb}|^2$ is the power of the sideband. We plot the CW steady-state solutions [1] to Supplementary Equation 1 and Supplementary Equation 2 in Supplementary Figure 4 (b) in red traces. Next we carry out numerical simulations based on the two independent equations with the same laser sweeping speed and range. We plot the corresponding $|A|^2_{\mathrm{max}}$ of the major and the minor states in Supplementary Figure 4 (b) in blue traces. One can see that the two sets of simulations agree very well in terms of the generated microcomb power and the detuning range for soliton microcomb existence. The discrepancy of the power fluctuations simulated with Eq. 1 in the main text around $\Delta_1 = 100\,\kappa$ indicates that the MI state of the minor comb introduces power instabilities of the major DKS state. Understandably, such interactions between the two comb states cannot be described by the independent LLEs, in spite of the qualitative agreement they yield.

## SUPPLEMENTARY NOTE 4: SOLITON BINDING MECHANISM

Conventionally the soliton binding mechanism is explained as the balance between the attractive and repulsive forces between two closely located solitons. Here we aim to provide an intuitive explanation on the physical mechanisms behind the forces, although a detailed quantitative analysis is beyond the scope of this work.

On the one hand, in the dissipative system with discrete drives, the major solitons and the minor solitons have different group velocities in the microresonator, which would close the temporal gap between the two solitons after

some time. On the other hand, as the two solitons are getting closer to each other, the field overlap between them starts to become significant. In monochromatically driven dissipative systems or in conservative systems, the relative phase between two adjacent solitons is fixed, therefore via the Kerr effect the field overlap provides an inter-soliton force, whose amplitude and direction depend on the inter-soliton separation. In other words, the field of one of the solitons functions as a potential well to exert force on the other. In our system, however, since the relative phase between the major soliton and the minor soliton is continuously rotating with a frequency of the sideband modulation frequency $\Omega$ (see Supplementary Figure 5), when the inter-soliton separation does not change, one may expect the time-averaged force due to relative-phase-related effects to vanish, which leaves the intensity-related effect, i. e. the cross phase modulation (XPM), to be the dominant mechanism.

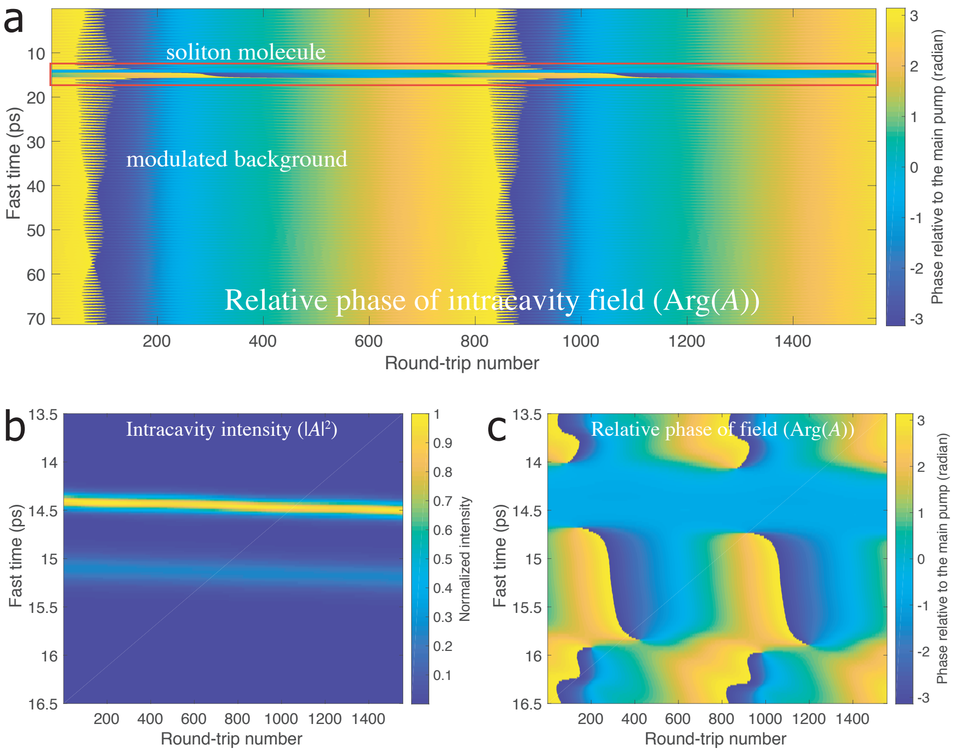


**Supplementary Figure 5**. **Phase evolution of heteronuclear soliton molecule.** (a) The evolution of the phase of intracavity field relative to the phase of the major pump field over two modulation periods (1556 round trips). The fast time locations of the major soliton and the minor soliton are around 14.4 ps and 15.6 ps respectively. (b) The map of the normalised soliton molecule field amplitude. (c) The blow-up of the phase evolution around the soliton molecule locations.

In our proof-of-principle simulation we treat the fields driven by the major and the minor pumps separately by using two individual LLEs coupled with XPM effect [2]. The coupled LLEs are written as:

$$\frac{\partial A_1}{\partial t} + i \sum_{j=2} \frac{D_j}{j!} (\frac{\partial}{i\partial \phi})^j A_1 - ig(|A_1|^2 + 2|A_2|^2) A_1 = \left(-\frac{\kappa}{2} + i(\omega_0 - \omega_{\mathrm{p}})\right) A_1 + \sqrt{\kappa_{\mathrm{ex}}} \cdot s_{\mathrm{in}} \tag{3}$$

$$\frac{\partial A_2}{\partial t} + i \sum_{j=2} \frac{D_j}{j!} (\frac{\partial}{i\partial \phi})^j A_2 - ig(|A_2|^2 + 2|A_1|^2) A_2 = \left(-\frac{\kappa}{2} + i(\omega_0 - \omega_{\mathrm{p}} - \Omega)\right) A_2 + \sqrt{\kappa_{\mathrm{ex}}} \cdot \frac{\epsilon}{2} s_{\mathrm{in}} \tag{4}$$

where $A_1$ and $A_2$ are the intracavity field envelopes of the major-pumped field and the minor-pumped field respectively. This model, of course, is a simplified model that neglects other phase-dependent coupling terms between the two fields. Here we note that such simplification can be justified due to the carrier frequency difference between the two distinct soliton fields. As can be seen from Supplementary Figure 5, the relative phase rotation period is much shorter than the time scale of the cavity photon decay time.

We simulate the evolutions of $A_1$ and $A_2$ based on the coupled LLEs with seeded solitons. Supplementary Figure 6 (a) and (b) show the results. In comparison with the simulation result based on a discretely driven single LLE (shown

in Supplementary Figure 6 (c)), one can see that the coupled LLEs yield almost identical soliton molecule formation process. In Supplementary Figure 6 (d) we also plot the intracavity field amplitudes at the end of the simulations when the heteronuclear soliton molecules are formed. The comparison shows that the inter-soliton separation derived by the coupled LLEs is slightly larger than that calculated with the single-LLE model. The discrepancy is mostly likely caused by the simplified coupling mechanism described by the coupled LLEs.

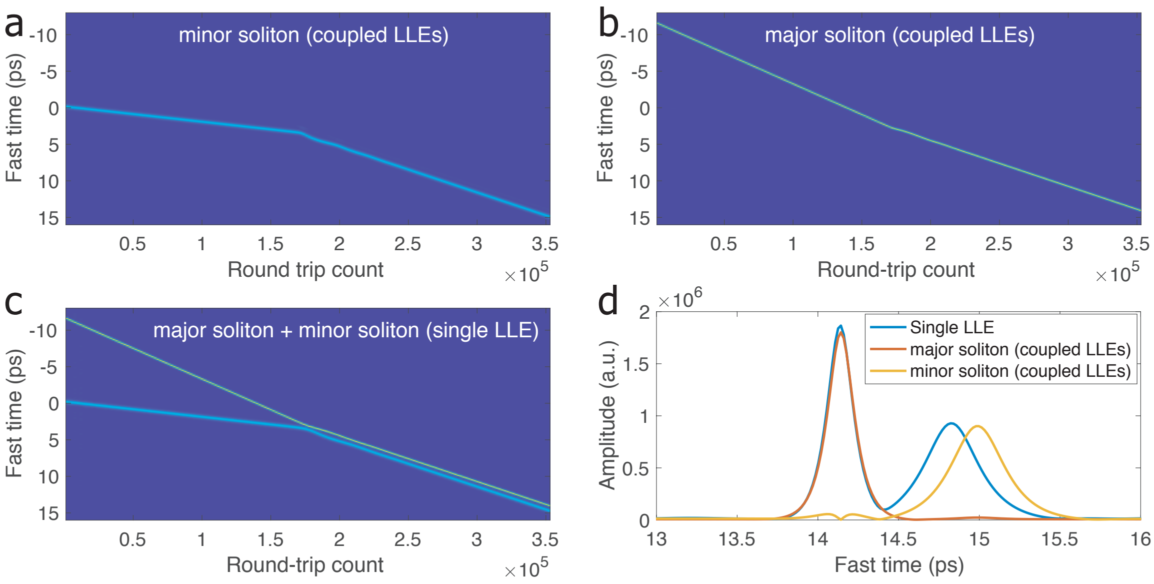


**Supplementary Figure 6**. **Comparison between single-LLE model and the model of coupled-LLEs.** (a, b) The evolutions of intracavity field envelopes that are driven by the minor pump and the major pump respectively. The solitons are seeded at the beginning of the simulation. At round trip number around $1.7 \times 10^5$ the two solitons meet and bind together, forming heteronuclear soliton molecule. (c) Simulation based on the single-LLE model. (d) The intracavity field amplitudes at the end of the simulations.

## SUPPLEMENTARY NOTE 5: COEXISTENCE OF SHORT- AND LONG-RANGE BINDING

For heteronuclear soliton molecules that are of multiple major or minor solitons, the situation is more complicated as the binding between similar solitons mostly relies on interlocking via dispersive waves. Indeed our simulations reveal that the coexistence of the short-range binding due to the XPM effect and the long-range binding caused by the dispersive-wave-mediated effect is essential for the formation of complex molecules beyond the basic form of single major soliton with single minor soliton. Supplementary Figure 7 (a) depicts the combined binding mechanism. Supplementary Figure 7 (b) shows the resonator dispersion used in the qualitative simulation. In order to introduce dispersive wave effect we add large frequency deviation to the resonance with a mode number of 60. As can be seen from the inset of Supplementary Figure 7 (b), such resonance frequency deviation generally leads to enhancement of the comb power in the mode, i. e. a single-mode dispersive wave. The simulation is started with a major soliton and two minor solitons seeded in the intracavity field. Supplementary Figure 7 (c) shows the evolution of the solitons. After the major soliton is bound with one of the minor solitons, the other minor soliton that is well separated from the bound pair also changes its soliton group velocity due to the long-range binding connecting the minor solitons. Consequently the three solitons travel with the same velocity, becoming a complex bound group. To stress the unique role of the dispersive wave, we repeat the simulation without the single mode resonance frequency deviation. As displayed in Supplementary Figure 7 (d), the simulation for comparison shows that without the long-range binding, the second minor soliton moves with its original velocity after the first minor soliton changes its velocity due to the binding with the major soliton. After some time, the two minor solitons collide into each other, leading to the annihilation of both.

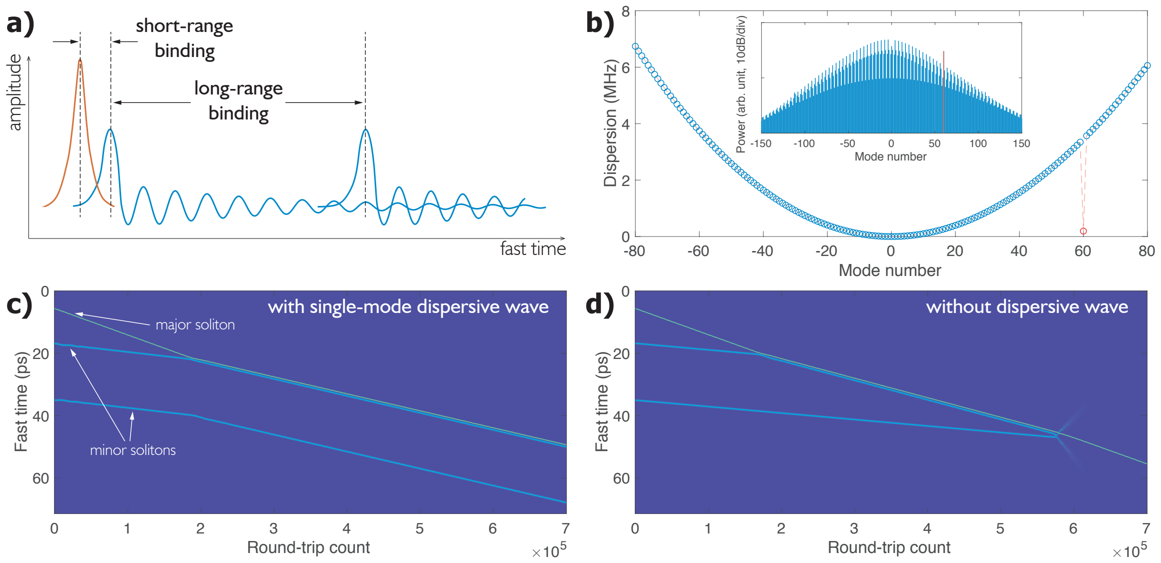


**Supplementary Figure 7**. **Coexistence of long- and short-range binding mechanisms.** (a) Schematic illustration of the coexistence of long-range (dispersive-wave-mediated) and short-range (XPM-induced) binding mechanisms. (b) Dispersion used in the simulation. At mode number of 60 the resonance frequency has a large deviation (shown in red circle) to produce intensive single-mode dispersive wave. The inset displays the comb spectrum, which shows the single-mode dispersive wave in red. (c) Simulated evolution of the bound state of 1 major soliton and 2 minor solitons with the dispersive-wave-mediated binding. (d) Simulated evolution of 1 major soliton and 2 minor solitons without introducing dispersive waves.

## SUPPLEMENTARY NOTE 6: REPETITION RATE OF DKS MOLECULE

Analytical expression of the repetition rate of DKS in optical microresonators with third-order dispersion has been derived with asymptotic analysis based on method of moments [3]. Here we use the results in that study to derive $f_{\text{rep}}$ of heteronuclear DKS molecules in resonators with third-order dispersion.

Based on the analysis in [3], when monochromatically pumped, the repetition rate shifts of the major and the minor DKS due to the third-order dispersion ($\Delta f_1$ and $\Delta f_2$) can be respectively expressed as:

$$\Delta f_1 = \frac{\delta_1 D_3}{3D_2} \tag{5}$$

$$\Delta f_2 = \frac{\delta_2 D_3}{3D_2} \tag{6}$$

where $\delta_1 = \frac{\omega_0 - \omega_{\text{p}}}{2\pi}$ and $\delta_2 = \delta_1 - \frac{\Omega}{2\pi}$ are the pump detunings respectively, and $D_2$ ($D_3$) is the second-order (third-order) dispersion coefficient.

When a heteronuclear DKS molecule is formed by a major soliton and a minor soliton, the constant velocity as well as the repetition rate shift of the DKS molecule ($\Delta f_3$) satisfy the conservation of soliton momentum, which can be written as:

$$E_3 \Delta f_3 = E_1 \Delta f_1 + E_2 \Delta f_2 \tag{7}$$

where $E_1$, $E_2$ and $E_3$ are the energies of corresponding solitons/molecules. With the approximation of the filed of solitons [3], soliton energy is proportional to the square root of the effective detuning ($E_{1,2} \propto \sqrt{\delta_{1,2}}$). Therefore the repetition rate shift of DKS molecules can be expressed as:

$$\Delta f_3 = \frac{E_1 \Delta f_1 + E_2 \Delta f_2}{E_1 + E_2} = \frac{D_3}{3D_2}(\delta_1 + \delta_2 - \sqrt{\delta_1 \delta_2}) \tag{8}$$

We perform numerical simulations to verify the theory derived above at three different major detunings ($90 \times \frac{\kappa}{2\pi}$, $110 \times \frac{\kappa}{2\pi}$ and $130 \times \frac{\kappa}{2\pi}$) with minor detuning varied from $20 \times \frac{\kappa}{2\pi}$ to $50 \times \frac{\kappa}{2\pi}$. We set the dispersion by using $\frac{D_2}{2\pi} = 2\,\text{kHz}$

and $\frac{D_3}{2\pi} = -4\,\mathrm{Hz}$. By fitting the motions of DKS molecules we derive $\Delta f_3$ of DKS molecules. The numerical results are presented in Supplementary Figure 8 in circles, triangles and diamonds. The theory are plotted in curves, showing good agreement. The increased discrepancy as the major detuning increases is attributed to the approximation used in [3].

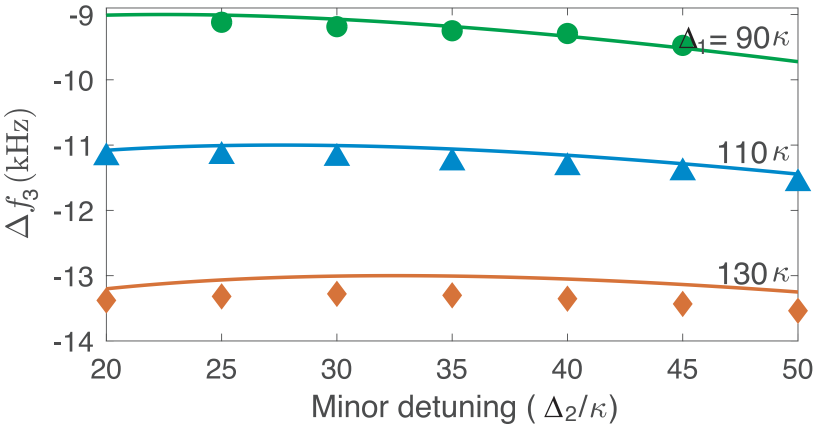


**Supplementary Figure 8. Numerical simulations (circles, triangles and diamonds) and theoretical calculations (curves) of the repetition rate shift of heteronuclear DKS molecules.** As denoted in the figure, three different major detunings of 90 $\kappa$, 110 $\kappa$ and 130 $\kappa$ are used for simulations respectively.

## SUPPLEMENTARY NOTE 7: FREQUENCY COHERENCE MEASUREMENT DETAILS.

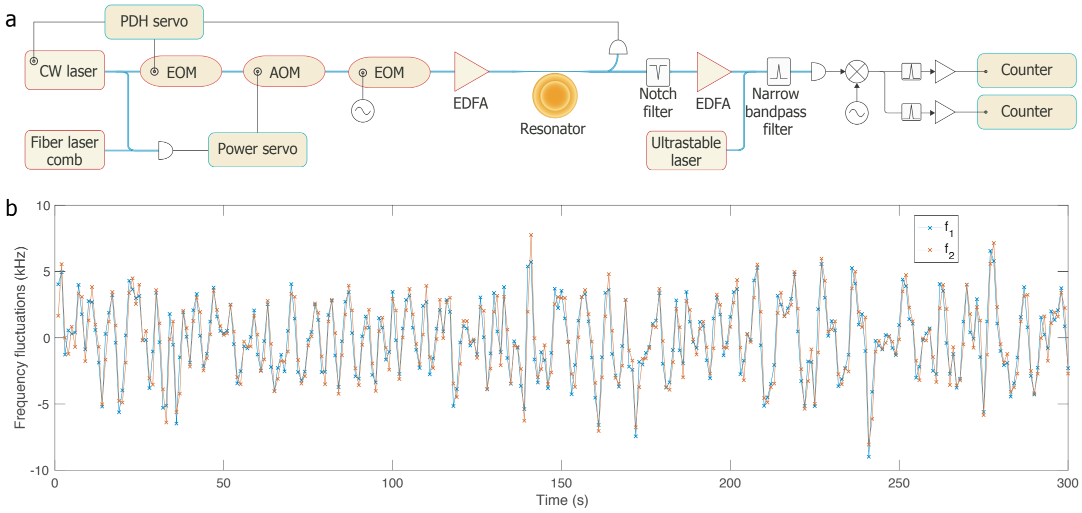


**Supplementary Figure 9. Experimental setup for frequency counting and the measured frequency fluctuations.** (a) The detailed setup for the stabilisation of the microcomb and the measurement of the frequency instabilities of a pair of comb teeth. (b) A fraction of simultaneously measured frequencies of the two beat signals $f_1$ and $f_2$. The modulation frequency $\Omega$ is removed from the frequency difference between $f_1$ and $f_2$, and an arbitrary frequency is subtracted from both $f_1$ and $f_2$ to set the data to around 0 kHz.

A 1553-nm laser whose frequency is stabilised to an ultrastable (relative frequency instability $< 1 \times 10^{-13}$ at integration time of 1 s) Fabry-Perot cavity to measure the frequencies of a pair of major and minor soliton comb teeth that is 20 FSRs ($\sim 2.3$ nm) apart from the pumped resonance. We also frequency-stabilise the superposed microcomb to a fully stabilised fibre-laser-based frequency comb by frequency-locking the effective detuning with Pound-Drever-Hall (PDH) locking technique and actively controlling the pumping power. The stabilisation setup is shown in Supplementary Figure 9 (a) and elaborated in [4]. We use two counters with a gate time of 1 s to measure the

two down-mixed beat frequencies ($f_1$ and $f_2$) at the same time and the recorded frequencies of the two signals allow us to confirm unambiguously that the frequency of the minor comb is offset from the frequency of the major comb by $\frac{\Omega}{2\pi}$. Supplementary Figure 9 (b) shows a small fraction (300 s) of the measured data. One should note that the offset frequency is subtracted from the difference between $f_1$ and $f_2$ in the data presented in Supplementary Figure 9 (b). To obtain the Allan deviations of averaging times $< 1$ s, a counter with gate time of 10 $\mu$s is used to measure the frequencies of $f_1$ and $f_2$ sequentially. The detuning locking bandwidth of $\sim 300$ Hz of the PDH locking system and the power control bandwidth of $\sim 0.5$ Hz limited mainly by the response of thermal expansion effect [5] are also shown in the two turning points at averaging times of 0.003 s and 2 s in the Allan deviation plot in the main text.

## SUPPLEMENTARY REFERENCES

[1] Coen, S. & Erkintalo, M. Universal scaling laws of kerr frequency combs. *Optics letters* **38**, 1790–1792 (2013).
[2] Agrawal, G. *Nonlinear Fiber Optics*. Optics and Photonics (Elsevier Science, 2012).
[3] Cherenkov, A., Lobanov, V. & Gorodetsky, M. Dissipative kerr solitons and cherenkov radiation in optical microresonators with third-order dispersion. *Physical Review A* **95**, 033810 (2017).
[4] Weng, W. *et al.* Spectral purification of microwave signals with disciplined dissipative kerr solitons. *Physical Review Letters* **122**, 013902 (2019).
[5] Weng, W. *et al.* Stabilization of a dynamically unstable opto-thermo-mechanical oscillator. *Physical Review A* **91**, 063801 (2015).
